# Epicardial adipose tissue thickness assessed by CT is a marker of atrial fibrillation in stroke patients

**DOI:** 10.1002/acn3.51617

**Published:** 2022-10-03

**Authors:** Fabian Edsen, Pardes Habib, Oliver Matz, Omid Nikoubashman, Martin Wiesmann, Michael Frick, Nikolaus Marx, Jörg B. Schulz, Arno Reich, João Pinho

**Affiliations:** ^1^ Department of Neurology University Hospital, RWTH Aachen University Aachen Germany; ^2^ JARA‐BRAIN Institute Molecular Neuroscience and Neuroimaging Forschungszentrum Jülich GmbH and RWTH Aachen University Aachen Germany; ^3^ Department of Diagnostic and Interventional Neuroradiology University Hospital, RWTH Aachen University Aachen Germany; ^4^ Department of Cardiology University Hospital, RWTH Aachen University Aachen Germany

## Abstract

Epicardial adipose tissue is involved in the pathophysiology of atrial fibrillation (AF). This study aimed to analyze its relevance as a stroke etiology marker. A retrospective study of acute ischemic stroke patients with large vessel occlusion was conducted, periatrial epicardial adipose tissue thickness (pEATT) on admission computed tomography angiography was measured. One hundred and twenty‐one patients with AF‐related stroke and 94 patients with noncardioembolic stroke were included. Patients with AF‐related stroke had increased pEATT. CT‐measured left‐sided pEATT was an independent predictor of AF‐related stroke (adjusted odds ratio per 1 mm increase = 1.27, 95% CI = 1.05–1.53, *p* = 0.012). pEATT is an independent marker of AF‐related stroke.

## Introduction

Periatrial epicardial adipose tissue (pEAT) is increasingly recognized to be associated with the presence of atrial fibrillation (AF) as well as its severity and recurrence.^1^ The mechanisms by which epicardial adipose tissue influences the atrial substrate for AF are not completely understood. It probably involves atrial adipose infiltration, secretion of inflammatory mediators, increased oxidative stress, promotion of fibrosis, and disruption of conduction.^2^ Recognition of markers associated with AF in patients with ischemic stroke may allow the stratification of patients with cryptogenic embolic stroke according to the likelihood of detecting an occult cardiac source such as paroxysmal AF.^3^ Our goal was to study pEAT thickness (pEATT) in acute stroke patients (AIS) with large vessel occlusion (LVO) and to assess its value in predicting the presence of AF.

## Methods

A single‐center retrospective study of prospectively collected consecutive AIS patients with LVO submitted to endovascular treatment (EVT) and available admission computed tomography angiography (CTA) covering the heart during an 18‐month period was conducted. Baseline, laboratorial, and echocardiographic characteristics were collected. Stroke etiology was reviewed and classified as noncardioembolic or AF‐related according to the Trial of Org 10172 in Acute Stroke Treatment (TOAST) classification. Routine stroke etiological investigations in our center included multimodal assessment of supra‐aortic and intracranial arteries, blood workup, transthoracic, and/or transesophageal echocardiography, ≥24‐h Holter electrocardiogram, continuous electrocardiogram monitoring with automated rhythm detection in patients admitted to the stroke unit or intensive care unit, and 24‐h blood pressure monitoring.

**Figure 1:**
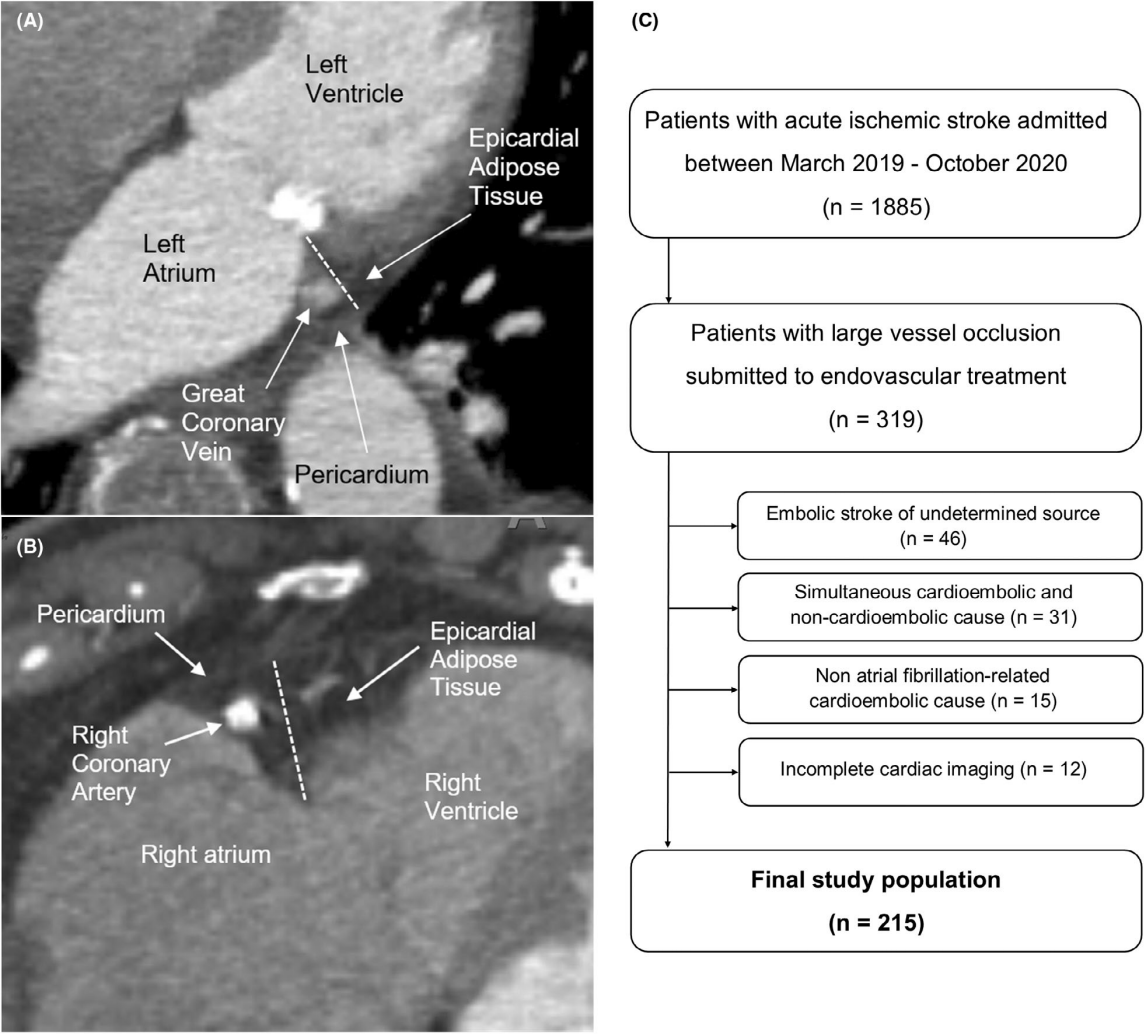
Measurement of periatrial epicardial adipose tissue on the left side (A) and on the right side (B). The distance between the angle of the atrioventricular groove and the visceral pericardium was measured in axial planes midway between the more superior and inferior atrial planes (dashed lines). Flow chart of patient selection (C).

Patients with simultaneous cardioembolic and noncardioembolic causes, non‐AF‐related cardioembolic causes, and embolic stroke of undetermined source (ESUS) were excluded. The category of noncardioembolic stroke included large vessel atherosclerotic disease and arterial dissection as well as other rare causes such as pro‐thrombotic state and carotid web. CTAs were performed on a multi‐detector CT scanner (SOMATOM Definition AS, Siemens, Erlangen, Germany) as a part of the emergency vascular diagnostic workup of acute stroke patients (collimation of 40 × 0.6 mm with 3 mm maximum intensity projection reformations in an intermediate kernel [B20f] and a reference tube–current–time product of 120 mAs). The field of view routinely included the supra‐aortic vessels as well as the heart. pEATT was measured in admission CTA in the axial plane by two independent observers blinded to stroke etiology (Fig. 1A and B). The mean value of the two measurements was used for the final analyses. Interobserver agreements were calculated with intraclass correlation coefficients (ICCs) for the consistency of individual measures. Baseline characteristics of patients with AF‐related stroke and noncardioembolic stroke were compared using the chi‐square test for categorical variables and the Mann–Whitney *U* test for continuous variables. Univariate and multivariable logistic regression analyses for the prediction of AF‐related stroke were performed separately for left‐ and right‐sided pEATT. Variables are known to influence the occurrence of AF (age, sex, arterial hypertension), possible relevant confounders (body mass index, low‐density lipoprotein [LDL] cholesterol, heart failure and/or left ventricle dysfunction, coronary heart disease), and other robust AF biomarkers (NTproBNP, severe left atrial enlargement) were selected as covariates in the models. All variables were included in the models in a single step. The threshold for statistical significance was set at an alpha value of 0.05. Retrospective studies based on the local registry of patients with AIS were approved by the local ethics committee, and patient informed consent was waived (approval reference 335/15). The data that support the findings of this study are available from the corresponding author upon reasonable request and according to local ethic guidelines.

## Results

Among 319 consecutive LVO AIS patients submitted to EVT during the study period, 215 patients were included in the final study population (Fig. 1C). Table 1 presents baseline characteristics according to stroke etiology. Among 121 patients with AF, the majority presented permanent AF (*n* = 57, 47.1%), 40 patients presented paroxysmal AF (33.1%), and 24 patients presented persistent AF (19.8%). Patients with AF were older, more frequently female, and had more frequent arterial hypertension, coronary heart disease, and heart failure. Among patients with AF, 76 (62.8%) patients had known AF prior to the stroke, therefore, AF‐related stroke patients were more frequently anticoagulated at baseline. Patients with noncardioembolic etiology more frequently smoked and presented higher levels of total cholesterol, non‐high‐density lipoprotein cholesterol, LDL cholesterol, and triglycerides. Patients with AF displayed more severe neurological deficits at admission, higher N‐terminal pro‐brain natriuretic peptide levels (NTproBNP), and a higher frequency of severe left atrial enlargement in echocardiography.

**Table 1:** Baseline characteristics of the study population according to the presence of AF‐related stroke or noncardioembolic stroke.

	Noncardioembolic stroke (n = 94)	AF‐related stroke (n = 121)	*p*
Male sex	67 (71.3)	41 (33.9)	<0.001
Age	66 (59–76)	81 (75–85)	<0.001
Body mass index	26.3 (24.7–29.4)	26.1 (24.4–29.4)	0.782
Comorbidities			
Arterial hypertension	71 (75.5)	106 (87.6)	0.021
Diabetes mellitus	35 (37.2)	39 (32.2)	0.444
Dyslipidemia	42 (44.7)	50 (41.3)	0.622
Coronary heart disease	17 (18.1)	40 (33.1)	0.014
Heart failure	4 (4.3)	21 (17.4)	0.003
Current smoking	31 (33.0)	10 (8.3)	<0.001
Metabolic syndrome	56 (59.6)	58 (47.9)	0.090
Chronic kidney disease	5 (5.3)	9 (7.4)	0.532
Chronic obstructive pulmonary disease	5 (5.3)	10 (8.3)	0.400
Baseline oral anticoagulation	1 (1.1)	55 (45.5)	<0.001
Anterior circulation large vessel occlusion	83 (88.3)	112 (92.6)	0.286
Admission National Institutes of Health Stroke Scale	10 (7–16)	14 (10–18)	0.041
Admission laboratory parameters			
Hemoglobin (g/dL)	13.2 (11.4–14.7)	12.2 (10.8–13.6)	0.008
Platelet count (/nL)	256 (214–285)	224 (186–275)	0.014
Admission glucose (mg/dL)	124 (105–170)	120 (108–148)	0.320
Total cholesterol (mg/dL)	152 (126–185)	144 (116–164)	0.035
LDL cholesterol (mg/dL)	102 (77–1299	83 (65–109)	0.004
HDL cholesterol (mg/dL)	38 (31–47)	42 (34–52)	0.061
Triglycerides (mg/dL)	152 (104–209)	103 (85–131)	<0.001
Non‐HDL cholesterol (mg/dL)	113 (86–142)	94 (74–120)	0.013
N‐terminal pro‐brain natriuretic peptide (pg/mL)^1^	285 (137–808)	1750 (992–3495)	<0.001
Echocardiography parameters^2^			
Severe left atrial enlargement^3^	0	15 (14.2)	<0.001
Left ventricle geometry			0.832
Normal	45 (57.0)	63 (58.9)	
Concentric remodeling	1 (1.3)	3 (2.8)	
Concentric hypertrophy	26 (32.9)	34 (31.8)	
Eccentric hypertrophy	7 (8.9)	7 (6.5)	
Left ventricle diameter			
End‐diastolic (mm)	47 (43–50)	47 (42–51)	0.640
End‐systolic (mm)	32 (28–35)	32 (27–37)	0.941
Left ventricle function			0.071
Normal	69 (86.3)	78 (73.6)	
Mild dysfunction	8 (10.0)	13 (12.3)	
Moderate dysfunction	3 (3.8)	10 (9.4)	
Severe dysfunction	0	5 (4.7)	
Periatrial epicardial adipose tissue thickness (mm)			
Left side	15 (14–17)	17 (15–20)	<0.001
Right side	16 (14–19)	18 (16–21)	0.001

^1^ Available for 188 patients.

^2^ Available for 186 patients.

^3^ Severe left atrial enlargement: defined subjectively by examiners; left atrial diameter ≥52 mm in men; ≥47 mm in women.

Interobserver agreement for pEATT was good (left side: ICC = 0.61, 95% CI = 0.52–0.68; right side: ICC = 0.65, 95%CI = 0.58–0.72). pEATT was positively correlated with age (left side: rho = 0.31, *p* < 0.001; right side: rho = 0.24, *p* < 0.001) and NTproBNP (left side: rho = 0.35, *p* < 0.001; right side: rho = 0.19, *p* = 0.003). Left‐sided pEATT was greater in patients with severe left atrial enlargement (median pEATT 19.5 vs. 16, *p* < 0.001) and in patients with known AF prior to stroke when compared to patients with newly diagnosed AF (median pEATT 17.5 vs. 16.5, *p* = 0.034).

In the univariate analysis, both left‐ and right‐sided pEATT were associated with AF‐related stroke. This association persisted only for left‐sided pEATT after adjustment for age, sex, body mass index, arterial hypertension, LDL cholesterol, coronary heart disease, heart failure and/or left ventricle dysfunction, severe left atrial enlargement, and NTproBNP (adjusted odds ratio per 1 mm increase = 1.27, 95%CI = 1.05–1.53, *p* = 0.012) (Table 2).

**Table 2:** Univariate and multivariate logistic regression analyses with atrial fibrillation‐related stroke as the dependent variable.

	Odds ratio (95% confidence interval)	*p*	Adjusted odds ratio^1^ (95% confidence interval)	*p*
Left‐sided pEATT^2^ (per 1 mm increase)	1.22 (1.11–1.33)	<0.001	1.27 (1.05–1.53)	0.012
Righ‐sided pEATT^2^ (per 1 mm increase)	1.13 (1.05–1.22)	0.002	1.00 (0.87–1.15)	0.998

^1^ Adjusted for age, sex, body mass index, arterial hypertension, low‐density lipoprotein cholesterol, heart failure and/or left ventricle dysfunction, coronary heart disease, severe left atrial enlargement, and N‐terminal pro‐brain natriuretic peptide.

^2^ Periatrial epicardial adipose tissue thickness.

## Discussion

This study demonstrates that among AIS patients with embolic stroke, increased pEATT on the left side is associated with the presence of AF. It may serve as a novel morphological marker for cardioembolism. Importantly, this association was independent of other well‐known markers of cardioembolism, namely NTproBNP and severe left atrial enlargement.

Several indirect markers of cardioembolic stroke have been studied, namely NTproBNP,^4^ left atrial enlargement,^5^ and reduced velocity in the left atrial appendage.^6^ Other markers such as frequent atrial premature complexes, P wave terminal force in V1, and atrial fibrosis in cardiac magnetic resonance imaging have also been associated with incident AF.^3^ The concept of atrial cardiopathy is based on these markers and it reflects the anatomical and functional atrial substrate for the development of AF.^7^ Our study expands the current body of literature concerning markers of atrial cardiopathy. The role of epicardial adipose tissue in the pathophysiology of AF is increasingly recognized.^1^, ^8^ In patients with AF undergoing pulmonary vein isolation, epicardial adipose tissue volume measured in CT is associated with AF recurrence.^9^ Additionally, the long‐term risk of embolic stroke in patients with AF submitted to catheter ablation is higher among patients with larger pEAT volume, and this association appears to be independent of AF recurrence.^10^ In another study of patients with persistent AF, the majority of whom were not anticoagulated, increased pEATT was also associated with an increased risk of cardiovascular events.^11^ A systematic review of 8 small studies has recently suggested an increased risk of stroke with increased epicardial adipose tissue, but there was marked heterogeneity in the study populations and methodologies.^12^ Only one previous study has analyzed epicardial adipose tissue in ischemic stroke patients with and without AF.^13^ It demonstrated that patients with AF‐related stroke had increased epicardial adipose tissue when compared to patients with ischemic stroke without AF, but this study displayed important limitations, namely measurement of epicardial adipose tissue only on the free wall of the right ventricle using echocardiography, the inclusion of non‐embolic stroke patients and absence of adjustment for important variables such as body mass index and NTproBNP. These limitations were addressed in the current study, which confirmed the independent association of epicardial adipose tissue and AF specifically in a group of patients with embolic ischemic stroke, thus expanding the literature on this subject.

As expected, the group of patients with noncardioembolic etiology had a higher frequency of vascular risk factors such as smoking and high cholesterol and triglyceride levels, which further highlights the different underlying pathophysiology of stroke in the two study groups. Epicardial adipose tissue is known to be significantly associated with the main obesity indices, but also with dyslipidemia, hypertension, and diabetes, and it is therefore also considered a modifiable risk factor for cardiovascular diseases.^14^, ^15^ Although our findings are not sufficient to induce a change in the current clinical practice, we believe that they are relevant for future stroke research concerning new markers for cardioembolism, by potentially improving the stratification of the risk of incident AF in ESUS patients. Limitations of our study include the relatively small sample size, the single‐center, retrospective nature of the study, and the absence of a systematic evaluation of echocardiographic parameters such as atrial strain. The fact that CTA protocol was not specifically performed to assess the heart, was not ECG‐triggered and the absence of 3D reconstruction measurements may be a source of bias, but allow simple implementation of a directly available structural assessment of the heart which does not delay emergent stroke care. The exclusion of only a few patients with no cardiac representation in admission CTA, few missing data and adjustment of our analyses for body mass index, and other robust markers for cardioembolism strengthen our results. Future research steps include the confirmation of these results in independent cohorts, the validation of pEATT as a potential marker for incident AF and/or stroke recurrence in ESUS patients, and to study the added value of including pEATT in the definition of atrial cardiopathy.

In conclusion, left‐sided pEATT is an independent marker for the presence of AF‐related stroke among AIS patients with large vessel occlusion.

## Conflict of interest

The authors report no conflict of interest.
